# Attribution of 2020 hurricane season extreme rainfall to human-induced climate change

**DOI:** 10.1038/s41467-022-29379-1

**Published:** 2022-04-12

**Authors:** Kevin A. Reed, Michael F. Wehner, Colin M. Zarzycki

**Affiliations:** 1 School of Marine and Atmospheric Sciences, Stony Brook University, Stony Brook, NY USA; 2 Applied Mathematics & Computational Research Division, Lawrence Berkeley National Laboratory, Berkeley, CA USA; 3 Department of Meteorology and Atmospheric Science, Pennsylvania State University, State College, PA USA

**Keywords:** Atmospheric dynamics, Environmental impact

## Abstract

The 2020 North Atlantic hurricane season was one of the most active on record, causing heavy rains, strong storm surges, and high winds. Human activities continue to increase the amount of greenhouse gases in the atmosphere, resulting in an increase of more than 1 °C in the global average surface temperature in 2020 compared to 1850. This increase in temperature led to increases in sea surface temperature in the North Atlantic basin of 0.4–0.9 °C during the 2020 hurricane season. Here we show that human-induced climate change increased the extreme 3-hourly storm rainfall rates and extreme 3-day accumulated rainfall amounts during the full 2020 hurricane season for observed storms that are at least tropical storm strength (>18 m/s) by 10 and 5%, respectively. When focusing on hurricane strength storms (>33 m/s), extreme 3-hourly rainfall rates and extreme 3-day accumulated rainfall amounts increase by 11 and 8%, respectively.

## Abstract (web summary)

During the 2020 hurricane season climate change increased the extreme rainfall rates and amounts by 5–10%.

## Introduction

Hurricanes are among the most costly and deadly geophysical extremes on Earth. Hurricane damage is caused by extreme wind speeds and flooding due to storm surge and heavy amounts of rainfall over relatively short periods of times. The recent 2020 North Atlantic hurricane season was a clear example of the destruction these storms can inflict on coastlines throughout North America and the Caribbean. The 2020 Hurricane season witnessed a record 30 named storms, 12 of which made landfall in the continental United States^1^, resulting in large regions experiencing hurricane rainfall. The total long-term economic costs from these storms are still being quantified, but estimates are over $40 billion^2^.

Hurricanes are fueled by energy and moisture associated with warm ocean temperatures. Increases in greenhouse gases in the atmosphere due to human emissions have resulted in a detectable increasing trend in global surface land and ocean temperatures over the last century attributable to human activities^3,4^. Quantifying the impacts of increasing sea surface temperatures (SSTs) on hurricanes, and tropical cyclones globally, remains a scientific challenge, given competing climate effects on other environmental parameters important for storm genesis and development, including wind shear and atmospheric stability^5^. However, a recent assessment by hurricane experts suggests that there has been an increase in intensity and the proportion of the most intense storms, as well as increase in the occurrence of storms resulting in extreme precipitation^6^. This assessment is supported by recent work that has shown evidence of increasing trends in intensity globally^7^.

Beyond studies of decadal trends, attribution frameworks in recent years have been developed to quantify the impact of human-induced warming on hurricane hazards, such as intensity and rainfall, for selected individual devasting storms. The use of hindcast attribution methodologies has demonstrated that human-induced warming has increased rainfall associated recent storms, including Irma and Maria in 2017^8^, Harvey in 2017^9^, Florence in 2018^10^, and Dorian in 2019^11^.

In this work, we apply the hindcast attribution methodology objectively to quantify the climate change impact on extreme rainfall throughout an entire North Atlantic hurricane season for 2020.

## Results

### Warming fingerprint

Human induced climate change has increased global average surface temperature by over 1 °C as of 2020^12^. This global-scale warming has altered the three-dimensional atmospheric states of temperature and moisture (e.g., specific humidity); thermodynamic quantities known to be relevant for hurricane development and formation^13,14^. The hindcast attribution methodology compares simulations of the actual world to conditional counterfactual simulations of a world without human interference in the climate system^15^ and is based on a pseudo-global warming framework^16^. Owing to a lack of global-scale preindustrial three-dimensional observational datasets, we construct the initial and boundary conditions defining this counterfactual world by altering observed 2020 conditions by a simulated anthropogenic warming fingerprint from a large climate model ensemble.

**Fig. 1:**
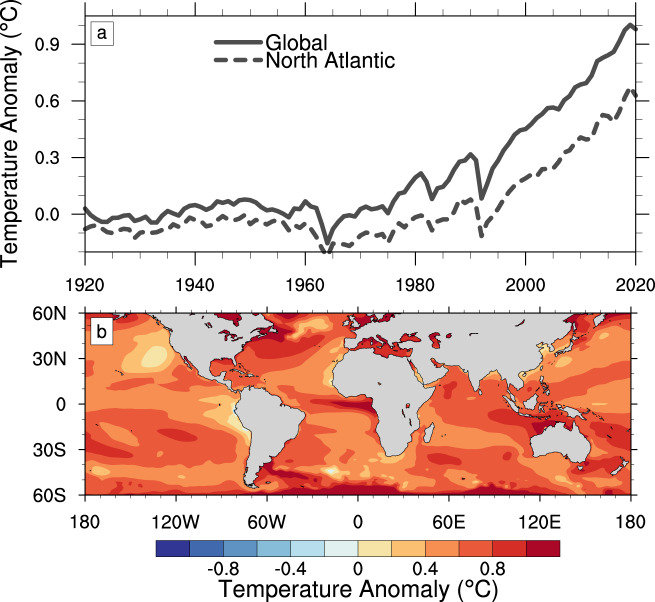
Global surface temperature change. **a** Evolution of the annual global and North Atlantic (defined to be 0–45°N) average surface temperature anomaly compared to 1850 using the CESM Large Ensemble from 1920 to 2020. **b** Global sea surface temperature anomaly for the 2020 North Atlantic hurricane season (June 1–November 30) relative to 1850 preindustrial period as calculated from the CESM Large Ensemble.

The Community Earth System Model (CESM) Large Ensemble^17^ is composed of a 1500-year 1850 control simulation and a transient simulation from 1850 onward. Starting in 1920 the initial transient simulation is used to initiate a 40-member ensemble to 2100 with the observed atmospheric composition through 2005 and Representative Concentration Pathway 8.5 boundary conditions afterwards. By averaging over the 40 member ensembles for each month and removing the corresponding 1850 control monthly average (for years 400–1500) we calculate a running estimate of the climate change fingerprint for each calendar month starting in 1920 for all thermodynamic variables. Note, by using the 1850 control simulation and Representative Concentration Pathway 8.5 forcing, the calculated anthropogenic fingerprint may contain a small solar forcing. Figure 1a shows the temporal evolution of the global average of this fingerprint for surface temperature and demonstrates that the global average temperature anomaly from the preindustrial period is approximately 1 °C in the CESM Large Ensemble for 2020, consistent with observations. A more comprehensive comparison of the CESM Large Ensemble simulated temperature evolution to observations is provided in previous work^17^.

### 2020 hindcast attribution simulations

**Fig. 2:**
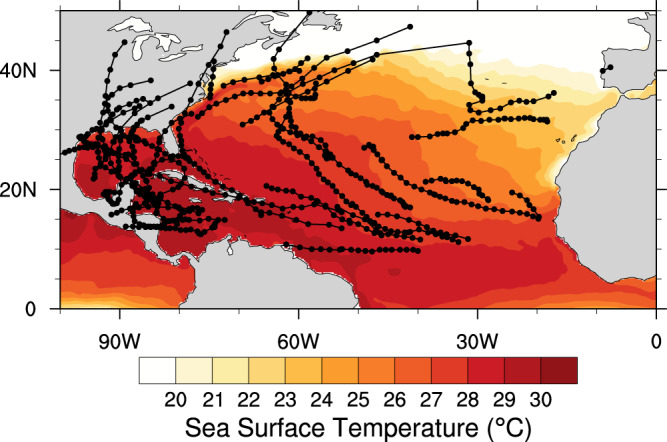
2020 hurricane tracks and surface temperature. Observed sea surface temperatures(color contours) and hurricane tracks for the 2020 North Atlantic hurricane season (June 1–November 30).

The 2020 North Atlantic hurricane season was extreme in that it produced 30 named storms. Of these storms, 29 occurred during the official hurricane season between June 1 and November 30, 2020 (Fig. 2). As expected, many of these storms occurred over the warm ocean temperatures of the tropical North Atlantic, where the average SST during the hurricane season was above 27 °C. As shown in Fig. 1b, it is estimated that the human influence on these 2020 SSTs using the CESM Large Ensemble was in the range of 0.4–0.9 °C throughout the majority of the North Atlantic, with an average of about 0.6 °C (Fig. 1a).

To quantify the impact of human-induced climate change on the rainfall associated with the 2020 Hurricane Season we run a series of hindcast simulations for the entire season following the forecast initialization procedure^18^ and hindcast attribution methodology^15^ with the Community Atmosphere Model (CAM), the atmospheric component of CESM. Week-long ensemble hindcasts are initialized every 3 days starting June 1 through November 30. Hindcasts that are initialized with atmospheric and ocean analyses are referred to as the “actual” ensemble since they are meant to simulate the actual conditions during the 2020 season. An additional ensemble of “counterfactual” hindcasts are also completed in which the surface boundary conditions are adjusted to remove the estimated anthropogenic SST fingerprint (Fig. 1b). The three-dimensional thermodynamic atmospheric variables of temperature and specific humidity are similarly adjusted at initialization using the CESM Large Ensemble and greenhouse gas concentrations are set to 1850 values.

**Fig. 3:**
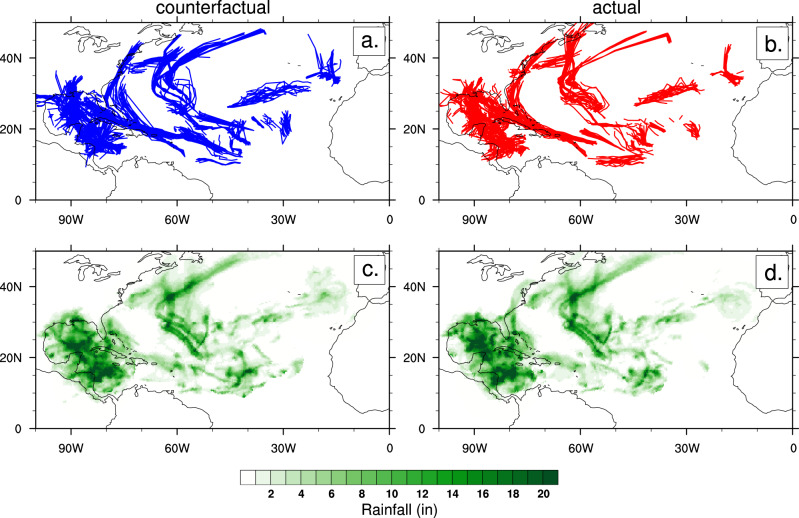
Actual and counterfactual storm tracks and rainfall. **a**, **b** Simulated storm tracks that match observed named storms and **c**, **d** ensemble average accumulated rainfall in inches (in) for the 2020 North Atlantic hurricane season (June 1–November 30) for the (**b**, **d**) actual and (**a**, **c**) counterfactual ensembles.

Figure 3(a, b) displays the simulated storm trajectories for all the counterfactual and actual ensembles for the entire 2020 hurricane season when they match an observed storm that is a tropical storm or stronger (winds greater than 18 m/s). As expected, there is spread in the ensembles, but given that 20-member ensembles are initialized every 3 days, there is overlap in the general distribution of tracks for the counterfactual and actual ensembles. The fact that the model is regularly constrained by observed conditions is an advantage of the hindcast attribution approach^10,11^ and allows for a comparison of the simulated storm rainfall.

The ensemble average accumulated rainfall for these storms is also displayed in Fig. 3(c, d). Since the individual ensemble members are 7-day hindcasts, there can be overlap in storm rainfall for events that last longer than 3 days (the time in between initializations). As a result, only 3 days of each ensemble member are included in the accumulated rainfall. Similar to the trajectories, the overall pattern of the simulated accumulated hurricane season rainfall is similar for the counterfactual and actual forecasts. There are subtle differences in certain regions, suggesting different amounts of rainfall for a given storm or slight deviations in ensemble trajectories.

### Attribution of hurricane season extreme rainfall

**Fig. 4:**
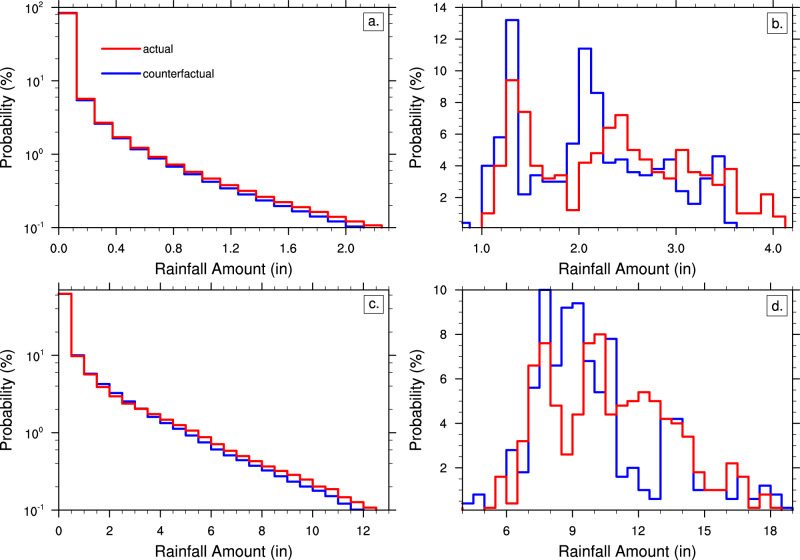
Changes in storm rainfall. Probability distributions of the **a**, **b** 3-hourly rainfall rate and **c**, **d** 3-day rainfall accumulated amounts in inches (in) associated with actual and counterfactual ensemble simulated storms during the 2020 hurricane season for observed storms of at least tropical storm strength. Results are shown for (left) for all rainfall output for all ensembles and (right) the 99th percentile amounts for each individual ensemble and initialization time. The sample size for **b**, **d** is 500.

Figure 4 shows the frequency distribution of all (Fig. 4a) 3-hourly storm rainfall rates and (Fig. 4c) 3-day accumulated storm rainfall amounts for the actual and counterfactual ensembles. While subtle, there are clear differences in the likelihood of both 3-hourly rates and 3-day accumulated amounts between the two ensembles. This is particularly true in the extremes of the distributions (i.e., 99th percentile).

Figure 4b shows the distribution of the 99th percentile storm rainfall rates for each of the individual ensemble members. While there is a large range in simulated 99^th^ percentile rates for each initialization and ensemble due to difference storm intensities at various times in their lifetime, there is a shift toward higher magnitudes in the actual ensemble compared to the counterfactual ensemble. This mean shift from 2.1 to 2.3 in. (~52 to 57 mm) in the 99th percentile of storm precipitation represents an 10.2% increase due to anthropogenic global warming, with a 95% confidence interval of 5.1 to 15.5%. Performing the same analysis for the 99^th^ percentile accumulated storm rainfall amount (Fig. 4d), reveals an anthropogenic increase in 3-day storm precipitation of 4.8% (95% confidence interval: 0.1 to 9.5%).

If we focus our analysis when observed storms are hurricane strength (surface winds greater than 33 m/s), the 99^th^ percentile hurricane rainfall rate increases from 2.3 to 2.6 in. (~59 to 66 mm), or about 11.3% (95% confidence interval: 6.3 to 16.5%). The change in the 3-day 99^th^ percentile accumulated hurricane rainfall amount 7.8% (95% confidence interval: 4.1 to 11.7%), which is noticeably larger than all storms of tropical storm strength or more.

## Discussion

This work quantifies the impact of climate change on rainfall throughout a full hurricane season. Using the CAM hindcast attribution methodology, human-induced climate change increased the extreme (defined to be the 99th percentile) 3-hourly storm rainfall rates and 3-day accumulated storm rainfall amounts during the 2020 hurricane season by 10% and 5%, respectively. This anthropogenic signal is even larger when focusing on storms that are at least hurricane strength, with increases in extreme 3-hourly hurricane rainfall rates and 3-day accumulated hurricane rainfall amounts of 11% and 8%, respectively. This increase in the anthropogenic signal in rainfall from hurricanes compared to rainfall from all tropical storm strength storms is an important finding with direct consequences for coastal communities.

The best estimate of the anthropogenic individual storm rainfall increases, here represented approximately by increases in 3-day accumulations, are consistent with Clausius-Clapeyron (C-C) scaling (~6–7%/C) given the estimated 0.4–0.9 °C anthropogenic warming in the North Atlantic basin. As storm accumulated rainfall is likely limited by available moisture, which in the highly saturated hurricane environment is controlled by C-C scaling, this result is not surprising. However, extreme 3-hourly storm rainfall increases at a rate best estimated at nearly twice C-C scaling, particularly for hurricane strength storms. We note that other work using hindcast attribution methodology at higher model spatial resolutions^8^ found structural changes in precipitation fields with localized total precipitation changes exceeding C-C expectations. As these simulated storms were intensified due to warming, their heaviest precipitating portions exhibited the largest percent rainfall increases. Such dynamical effects on storm structural characteristics could explain the increase in the tail of the hurricane rainfall distribution that we find here, but higher resolution simulations with full three-dimensional output are needed to explore these processes in more detail. Furthermore, while this work indicates that the thermodynamic anthropogenic fingerprint does result in increased rainfall rates and amounts in tropical cyclones, large-scale circulation impacts of climate change on storm frequency and development are not explored in the CAM hindcast attribution methodology.

This work objectively applies the hindcast attribution method to all storms of a given hurricane season, regardless of intensity or coastal impact. Most published work to date has focused, rightly so, on high impact, strong hurricanes with direct damages to coastlines and society. This work agrees with past work that has utilized various attribution frameworks on individual hurricanes to calculate the impact of climate change on storm rainfall to range from 2 to 20% depending on the rainfall metrics^8–11^. These changes in extreme rainfall associated with the North Atlantic hurricane season are an illustration of the likely climate change impacts on tropical cyclone rainfall in other ocean basins. This work is also consistent with a recent assessment of at least a “medium-to-high confidence” that an increase the projected future impacts of climate change on global tropical cyclone precipitation rates to be 7% per °C^19^, as well as a recent observational finding of a 1.3% global increase in tropical cyclone rainfall rate per year^20^. Other climate model-based estimates of the imposed pattern and magnitude of warming used to generate the counterfactual ensemble are equally credible but computational constraints preclude examining this source of uncertainty. However as presented here and in earlier studies, precipitation percent changes rather than absolute changes inform the scaling of hurricane precipitation as surface temperature changes. SSTs at both the global and regional scales will continue to increase in the coming decades due to human-induced greenhouse gas emissions. This work suggests that this warming will lead to yet further increases in North Atlantic hurricane season extreme rainfall rates and accumulated amounts. Finally, the application of hindcast attribution frameworks systematically to multiple storms throughout the hurricane season demonstrates the generalizability of such tools for operational climate change attribution applications beyond hurricanes.

## Methods

### CAM5 hindcast attribution method

This work makes use of the hindcast attribution method with the Community Atmospheric Model, version 5 (CAM)^10,11,15^. The CAM^21^ hindcasts use a variable resolution configuration with a high-resolution mesh with ~28 k grid spacing over the North Atlantic^22^. This high-resolution CAM grid has been shown to be sufficient in reproducing the North Atlantic hurricane climatology^23,24^, as well as storm-related rainfall for individual storms^10,11^ and climatological averages^25^. 20-member 7-day ensemble hindcasts are initialized every 3 days starting June 1 through November 30 following previous work^18^, resulting in 61 initialization times. Hindcasts that are initialized with NOAA atmospheric and ocean analyses from GDAS and OISST are referred to as the “actual” ensemble since they are meant to simulate the actual conditions during the 2020 season. An additional set of “counterfactual” ensemble hindcasts are also completed in which the seasonal average warming fingerprint for the three-dimensional thermodynamic atmospheric is removed from the initial conditions, the boundary conditions are adjusted to remove the SST fingerprint using the CESM Large Ensemble, and greenhouse gas concentrations are set to 1850 values. Volcanic aerosols and solar forcing are unchanged from the actual ensemble values. Note, that dynamical fields, including the zonal and meridional winds are not adjusted in the counterfactual hindcasts to avoid potential differences in storm trajectories between the ensembles. Both the actual and counterfactual simulations utilize a commonly-used digital filter to remove any hydrostatic imbalance associated with the initial state^18^. Finally, the ensemble members are generated using a perturbed parameter approach^10,11^. In total 1220 individual simulations were completed for both the counterfactual and actual ensembles.

### Storm detection and rainfall analysis

To extract precipitation associated only with tropical cyclones for each individual CAM ensemble we use the TempestExtremes software package^26,27^. TempestExtremes detects candidate cyclones and stitches together trajectories at 3-hourly increments. All analysis for this work only uses simulated storms while the observed storm is tropical storm strength with winds greater than 18 m/s. Furthermore, the simulated storms must initially be within 2 great circle degrees of an observed storm (but this does not have to occur at the initialization time of the ensemble), but after first detection is allowed to diverge by 5 great circle degrees of the observed later in its simulated lifetime. The total number of storms simulated is 1139 for the counterfactual ensemble and 1075 for the actual ensemble. TempestExtremes calculates an outer radius of azimuthally averaged azimuthal wind speed of 8 m/s within which all rainfall is defined to be due to the individual tropical cyclone^25^. The 99th percentile is then calculated from these storm-only precipitation fields for initializations in which all 20 individual ensemble members simulate an observed storm, which occurs for 25 of the 61 initialization times. All percentage differences and 95% confidence intervals between the actual and counterfactual ensembles are calculated using a bootstrap analysis of 10,000 samples.

## Data Availability

All raw CAM model output completed for this work is publicly available on the NCAR Globally Accessible Data Environment or is available on Google Drive (CEMSBU) by request due to the large data volume (>2TB). All CESM Large Ensemble output is publicly available via NCAR’s Climate Data Gateway [https://www.cesm.ucar.edu/projects/community-projects/LENS/data-sets.html]. All processed CAM storm track files are available on Zenodo [10.5281/zenodo.6046096]. All correspondence should be directed to K.A.R.

## Code availability

CAM code is available at www.cesm.ucar.edu and the Betacast (v0.1) code for initializing the hindcast is available on GitHub [https://github.com/zarzycki/betacast; 10.5281/zenodo.6047092].
