# Author Correction: Attribution of 2020 hurricane season extreme rainfall to human-induced climate change

**DOI:** 10.1038/s41467-022-30242-6

**Published:** 2022-05-05

**Authors:** Kevin A. Reed, Michael F. Wehner, Colin M. Zarzycki

**Affiliations:** 1 School of Marine and Atmospheric Sciences, Stony Brook University, Stony Brook, NY USA; 2 Applied Mathematics & Computational Research Division, Lawrence Berkeley National Laboratory, Berkeley, CA USA; 3 Department of Meteorology and Atmospheric Science, Pennsylvania State University, State College, PA USA

**Keywords:** Atmospheric dynamics, Environmental impact

Correction to: *Nature Communications* 10.1038/s41467-022-29379-1, published online 12 April 2022.

The original version of this Article contained an error in Fig. 1, in which Atlantic was spelt incorrectly. This has been corrected in both the PDF and HTML versions of the article.

